# The Association of Neighborhood Social Capital and Ethnic (Minority) Density with Pregnancy Outcomes in the Netherlands

**DOI:** 10.1371/journal.pone.0095873

**Published:** 2014-05-07

**Authors:** Vera L. N. Schölmerich, Özcan Erdem, Gerard Borsboom, Halleh Ghorashi, Peter Groenewegen, Eric A. P. Steegers, Ichiro Kawachi, Semiha Denktaş

**Affiliations:** 1 Erasmus University Medical Centre, Department of Obstetrics and Gynecology, Division of Obstetrics and Prenatal Medicine, Rotterdam, the Netherlands; 2 VU University Amsterdam, Department of Organizational Sciences, Amsterdam, the Netherlands; 3 Harvard School of Public Health, Department of Social and Behavioral Sciences, Boston, Massachusetts, United States of America; 4 Municipality of Rotterdam, Research and Business Intelligence, Rotterdam, the Netherlands; 5 Erasmus University Medical Center, Department of Public Health, Rotterdam, the Netherlands; 6 VU University Amsterdam, Department of Sociology, Amsterdam, the Netherlands; Old Dominion University, United States of America

## Abstract

**Background:**

Perinatal morbidity rates are relatively high in the Netherlands, and significant inequalities in perinatal morbidity and mortality can be found across neighborhoods. In socioeconomically deprived areas, ‘Western’ women are particularly at risk for adverse birth outcomes. Almost all studies to date have explained the disparities in terms of *individual* determinants of birth outcomes. This study examines the influence of neighborhood *contextual* characteristics on birth weight (adjusted for gestational age) and preterm birth. We focused on the influence of neighborhood social capital – measured as informal socializing and social connections between neighbors – as well as ethnic (minority) density.

**Methods:**

Data on birth weight and prematurity were obtained from the Perinatal Registration Netherlands 2000–2008 dataset, containing 97% of all pregnancies. Neighborhood-level measurements were obtained from three different sources, comprising both survey and registration data. We included 3.422 neighborhoods and 1.527.565 pregnancies for the birth weight analysis and 1.549.285 pregnancies for the premature birth analysis. Linear and logistic multilevel regression was performed to assess the associations of individual and neighborhood level variables with birth weight and preterm birth.

**Results:**

We found modest but significant neighborhood effects on birth weight and preterm births. The effect of ethnic (minority) density was stronger than that of neighborhood social capital. Moreover, ethnic (minority) density was associated with higher birth weight for infants of non-Western ethnic minority women compared to Western women (15 grams; 95% CI: 12,4/17,5) as well as reduced risk for prematurity (OR 0.97; CI 0,95/0,99).

**Conclusions:**

Our results indicate that neighborhood contexts are associated with birth weight and preterm birth in the Netherlands. Moreover, ethnic (minority) density seems to be a protective factor for non-Western ethnic minority women, but not for Western women. This helps explain the increased risk of Western women in deprived neighborhoods for adverse birth outcomes found in previous studies.

## Introduction

Despite free and high quality perinatal health care in the Netherlands, perinatal morbidity and mortality rates in this country remain relatively high compared to other European countries [1]. There are also large perinatal health inequalities between poor and wealthy urban neighborhoods [2]. In the second largest city, Rotterdam, neighborhood-specific preterm birth rates range from 34 to 153 per 1.000 births, and perinatal mortality ranges from 2 to 34 per 1000 births [3]. These are among the highest recorded disparities in birth outcomes across neighborhoods in any developed country.

On average, Western women show better birth outcomes than non-Western ethnic minority women, many of whom are first or second generation immigrants [4]. However, in 2008 a study indicated that in poor neighborhoods in the Netherlands, Western women appear paradoxically to be at higher risk for adverse birth outcomes compared to non-Western immigrant women [5]. These results were recently confirmed by a study on social deprivation and adverse perinatal outcomes among Western and non-Western pregnant women in Rotterdam [6].

Previous studies conducted in the Netherlands on birth outcome inequalities across neighborhoods and ethnic groups have mostly focused on *individual-level* determinants. Factors such as increased maternal age, non-Western ethnicity, and unhealthy lifestyle have been shown to be associated with adverse birth outcomes [7]. However, these individual factors cannot fully account for the between-neighborhood variation observed in birth outcomes. In other words, area-level disparities in birth outcomes are not purely attributable to compositional effects, i.e. the result of clustering of people with certain health characteristics in certain neighborhoods. There may be also contextual effects of neighborhood characteristics affecting health outcomes over and beyond the influence of individual determinants.

One study considered the effects of neighborhood income and deprivation on birth outcomes in Amsterdam, the largest Dutch city. This study only found ‘small-for-gestational age’ (SGA) to be associated with neighborhood income and deprivation [8]. Outside of the Netherlands, studies have found associations between a variety of neighborhood characteristics (including neighborhood socioeconomic status, social capital, and crime rate) and birth weight [9]–[11], preterm birth [12]–[14] and small-for-gestational-age [8], [15], [16].

### Neighborhood social capital, ethnic (minority) density and birth outcomes

An important source of resilience for residents of deprived neighborhoods is the level of ‘social capital’. The social capital of a neighborhood is measured by a) the extent of reciprocal exchanges between residents (i.e., the willingness of neighbors to help each other in times of need), b) the ability of residents to undertake collective action for mutual benefit (i.e., collective efficacy), c) the extent of social connections between members of a community, and d) trust. Trust is either seen as a component of social capital or as a result of social capital. Either way, trust is viewed as critical because without trust it is difficult to exchange favors or solve collective problems [17], [18]. For example, if **A** asks **B** to do a favor for her (e.g., look after her young children while she attends the prenatal clinic), **B** is more likely to agree to help if she trusts that **A** will repay the favor at a later date. Similarly, residents of a community are more likely to volunteer their time and effort to solve collective problems if they trust that their neighbors will also make an effort (as opposed to free-riding on the hard work of others). A neighborhood that is high in social capital is therefore one in which residents are constantly helping one another, with the result that some of the stresses associated with material disadvantage can be overcome or mitigated.

Past studies have repeatedly demonstrated an association between neighborhood social capital and adult morbidity and mortality [19]–[21]. Literature on neighborhood social capital and birth outcomes is scarce. Buka et al. and Morenhoff et al. found that neighborhood social capital is associated with higher birth weight [9], [10].

Another potentially relevant neighborhood attribute in the context of population health is the proportion of non-Western ethnic minority residents [22] – commonly referred to as ‘ethnic density’. In this study, we prefer to use the term ‘ethnic minority density ’. This is because the usage of the term ‘ethnic density’ reflects a limited definition of ethnicity – namely as a characteristic that only applies to minority groups, assuming that majority groups do not have any ethnicity.

In theory, neighborhoods with high ethnic minority density could exert divergent effects on the health of residents. On the one hand, a high spatial concentration of ethnic minorities could boost residents' sense of solidarity and cohesion, whilst minimizing contact with the majority group in society and thereby possibly reducing exposure to discrimination. This predicts that living in an area with high ethnic minority density might be protective for the health (and particularly the mental health) of residents. On the other hand, the presence of high ethnic minority density also suggests a spatial concentration of disadvantage (residential segregation and ‘ghettoization’). This may be harmful to health because of the lack of services and amenities, or the high prevalence of crime and other pathologies of poverty [22].

Studies of ethnic minority density and birth outcomes remain scarce and have found conflicting results. Some studies found that ethnic minority density was protective for certain ethnic minority groups for birth weight [16], [23] and preterm delivery [14], [16]. Other studies did not find ethnic minority density to be protective for ethnic minorities [9], [24], [25].

To our knowledge, all of the studies on neighborhood social capital and/or ethnic minority density on birth outcomes have been conducted in English-speaking countries, predominantly in the United States, Canada, and to a lesser extent the UK. However, as it has been argued by Poeran et al., the majority and minority groups in these countries are quite different to those in Europe in terms of ethnic origin and migration histories [6]. Moreover, the previous literature almost exclusively focuses on single urban populations, therefore using a much smaller individual and neighborhood sample size. Another limitation of the previous studies is that they often fail to adjust for all of the known relevant neighborhood level variables. Lastly, only one previous study by Buka et al. assessed the joint effect of ethnic minority density and neighborhood social capital with birth weight [9].

### Aims of this study

We sought to explore the association of neighborhood social capital and ethnic minority density with birth weight (adjusted for gestational age) and rates of premature births in the Netherlands. We assessed whether these associations persist after accounting for individual risks and other relevant neighborhood economic and environmental conditions. Lastly, we examined if neighborhood social capital and ethnic minority density can help to explain the increased risk of adverse birth outcomes among Western women living in deprived neighborhoods (compared to non-Western ethnic minority women).

## Methods

### Ethics and consent

The Perinatal Registration Netherlands committee approved this study. Written consent from pregnant women was not needed as the database protects their anonymity.

### Data sets

We combined four national data sets in the Netherlands on the basis of four-digit zip codes. Data on birth outcomes were obtained from the Netherlands Perinatal Registry, and data for neighborhood characteristics were derived from a) the Housing & Living Survey [26], b) the Netherlands Institute for Social Research, and c) Statistics Netherlands. These latter three data sets were open-source and based on survey and civil registration data. All of the data sets used in this study were nationally representative and covered the vast majority of inhabited four-digit zip codes areas (neighborhoods) in the Netherlands. The individual perinatal data was collected from 2000–2008, whilst the neighborhood characteristics were collected during 2005–2006.

**Figure 1:**
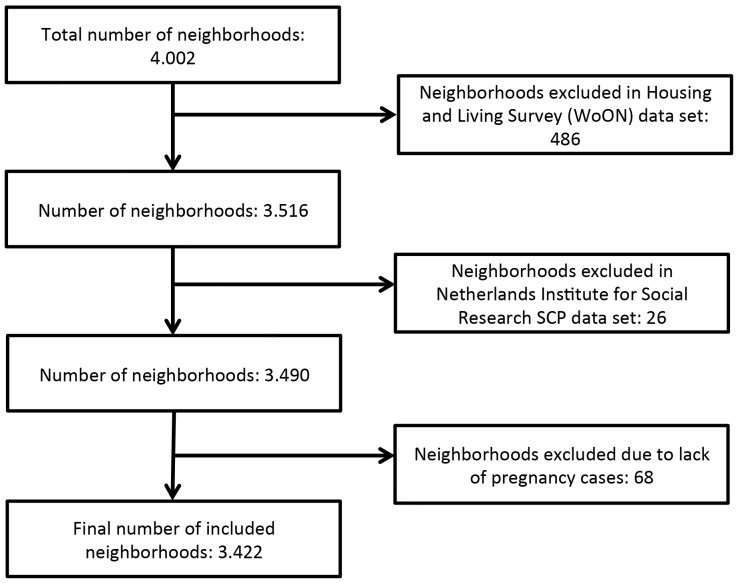
Exclusion of neighborhoods. Legend: This figure shows how many neighborhoods were excluded from the analysis due to missing data in the various data sets providing neighborhood characteristics.

**Figure 2:**
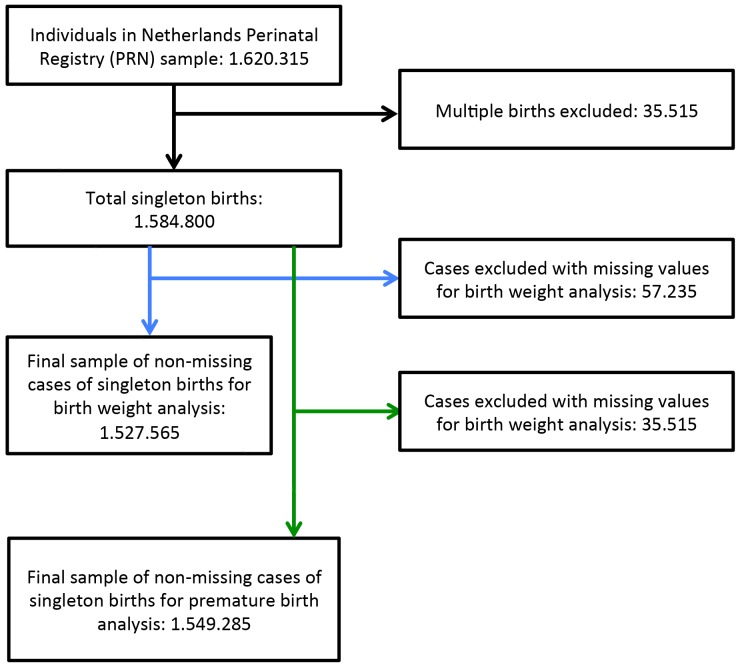
Exclusion of pregnancies. Legend: This figure indicates the number of pregnancies excluded from the birth weight and the premature birth analysis, respectively.

In our final analysis we included 3.422 neighborhoods and 1.527.565 and 1.549 285 singleton pregnancies for the birth weight analysis and the preterm birth analysis, respectively. Figure 1 shows the exclusion process for the neighborhoods. We excluded 580 neighborhoods (about 14% of total number of neighborhoods in the Netherlands) because not all of the six neighborhood characteristics used in our study were available for them. Most of these neighborhoods are industrial or rural areas with no or few residents. Figure 2 shows the exclusion process for the pregnancy cases. 57.235 pregnancies were excluded for the birth weight analysis and 35.515 for the premature birth analysis due to missing individual values (3,5% and 2,1% of total registered pregnancies, respectively).

Neighborhoods were defined as four-digit zip code areas. In 2006, the year that the data of the neighborhood characteristics was collected, an average of 4080 individuals lived in each four-digit zip code area. This geographical unit is comparable to the size of a US ‘census tract’ that is defined for the purpose of taking a census and often used in comparable studies. The four-digit zip code geographical unit we used is considered suitable for contextual studies in the Netherlands as they show enough sociocultural homogeneity [5].

### Individual characteristics

Data on singleton pregnancies were obtained from the Netherlands Perinatal Registry in the 2000–2008 dataset (www.perinatreg.nl). This registry contains 97% of all pregnancies in the Netherlands and has been collected by 94% of midwives, 99% of obstetricians, and 68% of pediatricians (including 100% of Neonatal Intensive Care Unit pediatricians). Descriptive statistics of the study population are provided in table 1. The two outcome measures are birth weight (in grams) and premature birth (gestational age before 37 weeks). We included the following maternal covariates for both analyses: maternal age, parity and ethnicity. For the birth weight analysis we also adjusted for sex of infant and gestational age.

**Table 1:** Descriptive statistics of individual variables and perinatal outcomes, source  =  Perinatal Registration Netherlands, 2000–2008.

	N	percentages
**Total singelton births**	1.584.800	97.80%
**Maternal age**		
<25 jr	188.795	11.9%
25–29 jr	456.742	28.8%
30–34 jr	621.528	39.2%
35–39 jr	276.944	17.5%
>40	40.791	2.6%
**Parity**		
Primiparous (first birth)	729.943	46.1%
Multiparous (second or higher birth)	854.424	53.9%
**Ethnicity**		
Western ethnicity	1.358.355	83.8%
Non-Western ethnicity	261.771	16.2%
**Sex infant**		
male	814.117	51.4%
female	769.959	48.6%
**Small-for-gestational-age (SGA)** *	152.848	9.6%
**Premature births (<37 week of gestation)**	97.353	6.1%
**Birthweight in grams (mean, SD)**	3446.81	594.3 (SD)

*SGA =  birth weight below 10th percentile for gestational age (Kloosterman, 1970).

We focus on low birth weight (adjusted for gestational age) and preterm births as these are the two most prevalent forms of perinatal morbidity and also the two most important predictors of perinatal mortality in the Netherlands. Low birth weight and preterm birth are also associated with important adverse physical and psycho-social long-term effects [27]–[29].

#### Ethnicity

Dutch law does not permit the routine utilisation or registration of ethnic origin in clinical settings. As yet, The Netherlands Perinatal Registry is exempt from this restriction. The classification of the Netherlands Perinatal Registry defines ‘ethnicity’ along seven categories: Western Dutch, Western other (including women from other European countries, Australia, and the US), Mediterranean, (East) Asian, African, South Asian, or other non-Western. The African and South Asian groups are mainly composed of women from the former Dutch colonies Suriname and the Netherlands Antilles. The group of East Asian women mainly originates from Indonesia, which is also a former Dutch colony. The classification of ethnicity is made by the healthcare professional. This method of registering ethnicity is problematic in several ways (see ‘discussion/limitations’). From a methodological standpoint, it is likely to produce classification error. As we were primarily interested in examining why Western women are at higher risk for adverse perinatal outcomes in poor neighborhoods, we opted for a crude binary classification of Western versus non-Western ethnic minority women. To do this, we defined the first two classes of the original classification as “Western” women and the other five classes were together defined as “non-Western ethnic minority” women. By collapsing into these simplified two categories, we sought to circumvent the misclassification introduced by the method of ethnicity ascertainment on the registry records. Another advantage of this dichotomy is that is makes the results comparable to previous studies [5], [6]. The limitations of such a dichotomous representation will be discussed later.

### Neighborhood characteristics

We included neighborhood characteristics that have been shown to be associated with birth weight and/or preterm births (social capital, ethnic minority density, socioeconomic status and feeling of safety in neighborhood). Two other characteristics that have been shown to influence general health outcomes were also included (urbanity of neighborhood and home maintenance) [20]. The correlations of the neighborhood-level variables are indicated in table 2.

**Table 2:** Correlations of neighborhood variables.

nj = 3422	1	2	3	4	5	6
**1. Ethnic minority density**	1	-	-	-	-	-
**2. Neighborhood social capital**	−,565**	1	-	-	-	-
**3. Socio-economic status**	−,562**	,346**	1	-	-	-
**4. Urbanity**	,588**	−,505**	−,237**	1	-	-
**5. Home maintenance**	−,281**	,278**	,323**	−,183**	1	-
**6. Feeling of safety**	−,412**	,385**	,293**	−,320**	,264**	1

*p≤0.05,

**p≤0.01,

***p≤0.001.

nj =  numbers of neighborhoods.

#### Neighborhood home maintenance

(data source: Housing & Living Survey). We used home maintenance as a proxy for the environmental condition of a neighborhood. Maintenance was assessed with the question “Is your house in a bad condition?” Answer categories were on a 5-point scale from ‘I totally agree’ (1) to ‘I totally do not agree’ (5). Higher values thus indicate better maintenance.

#### Urbanity

(data source: Housing & Living Survey). This variable indicates the degree of urbanity of the municipality of a neighborhood, measured by numbers of addresses per km2: 5) urban, more than 2499 addresses/km2; 4) semi-urban, 1500–2499 addresses/km2; 3) intermediate urban-rural, 1000–1499 addresses/km2; 2) semirural, 500–999 addresses/km2; 1) rural, up to 499 addresses per km2.

#### Feeling of safety

(data source: Housing & Living Survey). This variable was addressed with the statement “I am scared of being harassed or assaulted in this neighborhood”. Answer categories were on a 5-point scale from ‘I totally agree’ (1) to ‘I totally do not agree’ (5). Higher values thus indicate higher feeling of safety.

#### Socioeconomic status

(data source: Netherlands Institute for Social Research). This is a composite measure for socioeconomic status. It provides information on average income, the percentage of people with low income, a low education, and the percentage of unemployed. A higher score indicates higher socioeconomic status.

#### Ethnic minority density

(data source: Statistics Netherlands). This variable provides the percentages of different non-Western ethnic minority people per neighborhood. For the purpose of this study, we grouped together the non-Western ethnic minority groups into the ‘non-Western’ category, as was done for the perinatal registry data (see section above ‘ethnicity’).

#### Neighborhood social capital

(data source: Housing & Living Survey). We used five items to construct this scale:

- contact with direct neighbors
- contact with other neighbors
- whether people in the neighborhood know each other
- whether neighbors are friendly to each other
- whether there is a friendly and sociable atmosphere in the neighborhood

Response categories were on a 5-point Likert scale from ‘I totally agree’ (1) to ‘I totally do not agree’ (5) and were coded in such a way that higher values indicate higher social capital. We applied the ‘ecometrics’ methodology in order to calculate a neighborhood social capital score for each neighborhood (see the following section). More detailed information about the data sets is provided as supporting information (Table S1).

### Ecometrics

We used an ecometrics analysis in order to aggregate social capital items at the individual level to the neighborhood level [30], [31]. As Mohnen et al. (2011) outline, this approach accounts for: 1) between-neighborhood differences in individual characteristics that influence responses to items, 2) differences in numbers of respondents per neighborhood, and 3) nesting of the items within individuals (dependency between items on the individual level) as well as individuals within neighborhoods [20].

To calculate the neighborhood social capital score, we used a linear multilevel model with three levels: items, individuals, and neighborhoods. This 3-level analysis allowed us to take the nesting of social capital items within individuals and neighborhoods into account. The five items measuring social capital formed the dependent variables. The model was adjusted for eight individual characteristics that may influence respondents' perception of social capital: sex, age, ethnicity, education, income, employment status, home ownership, and years of residence in neighborhood. The variation in numbers of respondents per neighborhood is accounted for in the model by shrinking deviating neighborhoods with smaller number of respondents to the general average [32].

The model used is as follows: 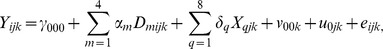



*Y_ijk_* is the response of item i of respondent j in neighborhood k, 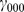 is the grand mean of neighborhood social capital, *m* is the number of social capital variables (5, one being the reference category), *D* are item dummies, *q* is the number of individual-level adjusters (8), *X* are the adjusters, *v* denotes the neighborhood variance, *u* denotes individual variance and *e* denotes item variance. The neighborhood level residuals (*v*) from this model constitute the neighborhood social capital scores that are then used in the main analysis of this study (see below), with a higher value indicating a higher level of neighborhood social capital.

In this analysis, an average of 18,3 individuals were nested within a total of 3.495 neighborhoods. The reliability of ecometric calculations depends on the variance at all three levels [32]. Based on the estimator found in Hox [32], the reliability of our neighborhood social capital scale is acceptable at 0.595. This value can be interpreted in a similar manner as Cronbach's alpha in psychometrics. Finally, the correlation between the straightforward aggregated measure of neighborhood social capital and that derived with the ecometrics approach is 0.77. The ecometrics analysis was performed using MLwiN 2.02.

### Analytical strategy

We performed two separate multi-level analyses: a linear regression for birth weight (in grams) and a logistic regression for preterm birth defined as <37 weeks of gestation (0 = not preterm, 1 = preterm). We performed seven model specifications following the same pattern for both analyses. First, we estimated an empty model including only a random intercept for neighborhoods to assess the clustering of the outcome across neighborhoods. Then we sequentially added the individual and neighborhood characteristics as fixed effects. The seven models are presented in table 3 and 4. The interaction terms non-Western ethnicity*neighborhood social capital and non-Western ethnicity*ethnic minority density are included in the fifth and sixth models. Model 7 shows the full model with all individual and neighborhood level variables. We plotted the interaction terms for ethnicity and birth weight to further assess this result. In addition to these main analyses, we ran the same analysis as for birth weight using ‘small-for-gestational-age’ as the outcome, defined as birth weight below the 10th percentile for gestational age. Moreover, we also ran the main analyses using the aggregated neighborhood social capital score. All of these analyses were performed in SPSS 20.

**Table 3:** Multilevel regression models of neighborhood social capital and ethnic density on birth weight.

ni = 1.527.565	nj = 3422	Model 1		Model 2		Model 3		Model 4		Model 5		Model 6		Model 7	
Intercept		3667.1	(3664.5/3669.8)***	3664.1	(3661.4/3666.8)***	3661.5	(3658.8/3664.2)***	3661.9	(3659.2/3664.5)***	3661.2	(3658.5/3663.9)***	3659.9	(3657.3/3662.6)***	3678,0	(3657.3/3662.6)***
***Individual level***															
Maternal age (Ref. = 25–29 yrs)	<25 jr	−44.6	(−47.4/−41.9)***	−44.2	(−47.0/−41.4)***	−44.0	(−46.8/−41.2)***	−43.4	(−46.2/−40.6)***	−43.3	(−46.1/−40.5)***	−43.1	(−45.9/−40.3)***	−43.1	(−45.9/−40.3)***
	30–34 jr	9.8	(7.8/11.7)***	9.9	(7.9/11.9)***	9.7	(7.7/11.6)***	9.5	(7.5/11.5)***	9.5	(7.6/11.5)***	9.6	(7.6/11.5)***	9.6	(7.6/11.5)***
	35–39 jr	−3.9	(−6.3/−1.4)***	−3.7	(−6.2/−1.2)***	−3.8	(−6.3/−1.4)***	−4.2	(−6.7/−1.7)***	−4.1	(−6.6/−1.6)***	−4.0	(−6.5/−1.5)**	−4.0	(−6.4/−1.5)**
	>40	−35.7	(−40.8/−30.5)***	−35.2	(−40.4/−30.1)***	−35.5	(−40.6/−30.3)***	−35.6	(−40.7/−30.4)***	−35.4	(−40.6/−30.2)***	−35.2	(−40.3/−30.0)***	−35.1	(−40.3/−30.0)***
Parity (Ref. = multiparous)	primiparous	−150.3	(−152.0/−148.6)***	−150.3	(−152.0/−148.7)***	−150.2	(−151.9/−148.6)***	−150.4	(−152.1/−148.7)***	−150.2	(−151.9/−148.5)***	−150.0	(−151.7/−148.4)***	−150.0	(−151.7/−148.3)***
Ethnicity (Ref. = Western)	non-Western	−87.1	(−89.5/−84.6)***	−85.6	(−88.1/−83.2)***	−83.4	(−85.9/−81.0)***	−83.4	(−85.9/−80.9)***	−88.8	(−91.5/−86.1)***	−91.7	(−94.4/−89.0)***	−92.3	(−95.0/−89.5)***
Sex infant (Ref. = male)	female	−133.8	(−135.3/−132.2)***	−133.8	(−135.4/−132.3)***	−133.9	(−135.4/−132.3)***	−133.9	(−135.4/−132.3)***	−133.9	(−135.4/−132.3)***	−133.9	(−135.4/−132.3)***	−133.9	(−135.4/−132.3)***
Gestational age (Ref. = ≥37 wks)	<37 weeks	−1249.3	(−1252.4/−1246.2)***	−1249.1	(−1252.3/−1246.0)***	−1249.3	(−1252.4/−1246.2)***	−83.4	(−85.9/−80.9)***	−88.8	(−91.5/−86.1)***	−91.7	(−94.4/−89.0)***	−92.3	(−95.0/−89.5)***
***Neighborhood level***															
Ethnic minority density						−22.0	(−24.4/−19.5)***	−7.4	(−11.2/−3.6)***	−8.6	(−12.4/−4.8)***	−15.6	(−19.5/−11.6)***	−15.2	(−19.1/−11.2)***
Neighborhood social capital				11.6	(9.513.7)***			1.2	(−1.5/3.8)	2.7	(0.1/5.4)*	0.3	(−2.3/2.9)	0.8	(−1.9/3.4)
Socio-economic status								16.5	(13.8/19.2)***	16.5	(13.8/19.2)***	16.3	(13.6/19.0)***	16.3	(13.6/19.0)***
Urbanity								0.6	(−1.7/2.9)	1.2	(−1.2/3.5)	2.2	(−0.2/4.5)	2.1	(−0.2/4.5)*
Home maintenance								−2.0	(−3.8/−0.1)*	−2.0	(−3.8/−0.1)*	−2.0	(−3.9/−0.2)*	−2.0	(−3.9/−0.2)*
Feeling of safety								3.5	(1.5/5.6)***	3.5	(1.5/5.6)***	3.4	(1.4/5.5)***	3.5	(1.4/5.5)***
Ethnic minority density*non-Western												16.4	(14.2/18.5)***	15.0	(12.4/17.5)***
Neighb. social capital*non-Western										−12.3	(−14.8/−9.9)***			−2.8	(−5.7/0.1)

(coefficients, 95% confidence intervals in parentheses).

*ni = number of individuals; nj =  number of neighborhoods*.

*p≤0.05,

**p≤0.01,

***p≤0.001.

**Table 4:** Multilevel logistic regression models of neighborhood social capital and ethnic density on premature birth.

ni = 1.549.285	nj = 3422	Model 1		Model 2		Model 3		Model 4		Model 5		Model 6		Model 7	
Intercept		0.05	(0.05/0.06)***	0.05	(0.05/0.06)***	0.05	(0.05/0.05)***	0.05	(0.05/0.05)***	0.05	(0.05/0.05)***	0.05	(0.05/0.06)***	0.05	(0.05/0.06)***
***Individual level***															
Maternal age (Ref. = 25–29 yrs)	<25 jr	0.99	(10.97/1,01)	0.99	(0.97/1.01)***	0.98	(0.96/1.00)	0.98	(0.96/1.00)*	0.98	(0.96/0.99)*	0.98	(0.96/1.00)*	0.98	(0.96/1.00)*
	30–34 jr	1.03	(1.06/1.06)***	1.03	(1.02/1.05)***	1.03	(1.02/1.05)***	1.04	(1.02/1.05)***	1.04	(1.02/1.05)***	1.04	(1.02/1.05)***	1.04	(1.02/1.05)***
	35–39 jr	1.14	(1.12/1.16)***	1.14	(1.12/1.16)***	1.14	(1.12/1.16)***	1.15	(1.13/1.17)***	1.15	(1.13/1.17)***	1.15	(1.13/1.17)***	1.15	(1.13/1.17)***
	>40	1.41	(1.36/1.46)***	1.41	(1.36/1,47)***	1.41	(1.36/1.46)***	1.42	(1.36/1.48)***	1.42	(1.37/1.48)***	1.42	(1.37/1.47)***	1.42	(1.37/1.48)***
Parity (Ref. = multiparous)	primiparous	1.78	(1,76/1,81)***	1.78	(1.76/1.80)***	1.78	(1.76/1.81)***	1.79	(1.77/1.81)***	1.79	(1.77/1.81)***	1.79	(1.76/1.81)***	1.79	(1.76/1.81)***
Ethnicity (Ref. = Western)	non-Western	1.03	(1,01/1,05)***	1.04	(1,01/1,05)***	1.02	(1.00/1.04)	1.02	(0.98/1.04)	1.02	(1.00/1.04)***	1.03	(1.01/1.05)**	1.03	(1.01/1.05)**
***Neighborhood level***															
Ethnic minority density						1.03	(1.02/1.04)***	1.03	(1.01/1.05)***	1.03	(1.02/1.05)***	1.04	(1,03/1,06)***	1.05	(1.03/1.06)***
Neighborhood social capital				0.99	(0,98/01.00)			0.99	(0.98/1.00)	0.99	(0.98/1.00)	0.99	(0.98/1.01)	1.00	(0.98/1.01)
Socio-economic status								0.95	(0.94/0.97)***	0.95	(0.94/0.97)***	0.95	(0.94/0.97)***	0.95	(0.94/0.97)***
Urbanity								0.95	(0.94/0.96)***	0.95	(0.94/0.96)***	0.95	(0.94/0.96)***	0.95	(0.94/0.96)***
Home maintenance								1.02	(1.00/1.03)**	1.02	(1.01/1.03)**	1.02	(1.01/1.032)***	1.02	(1.01/1.03)***
Feeling of safety								0.99	(0,98/1,00)	0.99	(0.98/1.00)	0.99	(0.98/1.00)	0.99	(0.98/1.00)
Ethnic minority density*non-Western												0.98	(0.96/0.99)***	0.97	(0.95/0.99)***
Neighb. social capital*non-Western										1,01	(0.99/1.03)			0.99	(0.97/1.01)

(Odds ratios, 95% confidence intervals in parentheses).

*ni = number of individuals; nj =  number of neighborhoods*.

*p≤0.05,

**p≤0.01,

***p≤0.001.

The intraclass correlation for the logistic models was calculated using the following formula [33]: 
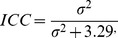



## Results

The average birth weight of infants in the Netherlands from 2000–2008 is 3446.8 grams (SD = 594.3), and the prevalence of preterm births is 6.1%. More detailed descriptive statistics on the population are given in table 1. As can be seen in tables 3 and 4, the estimates for all individual-level variables are in the expected direction and remain relatively stable across the models. Women who are under 25 or above 40 years of age, primiparous, and who belong to a non-Western ethnic minority group, tend to have infants with a lower weight and a higher risk for preterm birth. Moreover, female infants are likely to have lower birth weight.

Table 2 indicates that neighborhoods with higher socioeconomic status tend to have lower ethnic minority density (corr. −0,57, p<0.001) and higher neighborhood social capital (corr. 0.35, p<0.001). The empty models of both regression analyses (results not shown) indicate that average birth weight and risk for preterm birth varies significantly across neighborhoods (1.0% and 0.7%, respectively, results not shown). The results of the regression models are shown in table 3 and 4. Even after controlling for individual compositional characteristics, we found a small but significant clustering of birth weight outcomes and prevalence of prematurity within neighborhoods.

Model 3 of both regression models (table 3 and 4) shows that ethnic minority density is associated with a decrease in birth weight and an increase in risk for preterm births. As seen in models 4, the effect of ethnic minority density remains significant when controlling for other relevant neighborhood characteristics for both analyses.

**Figure 3:**
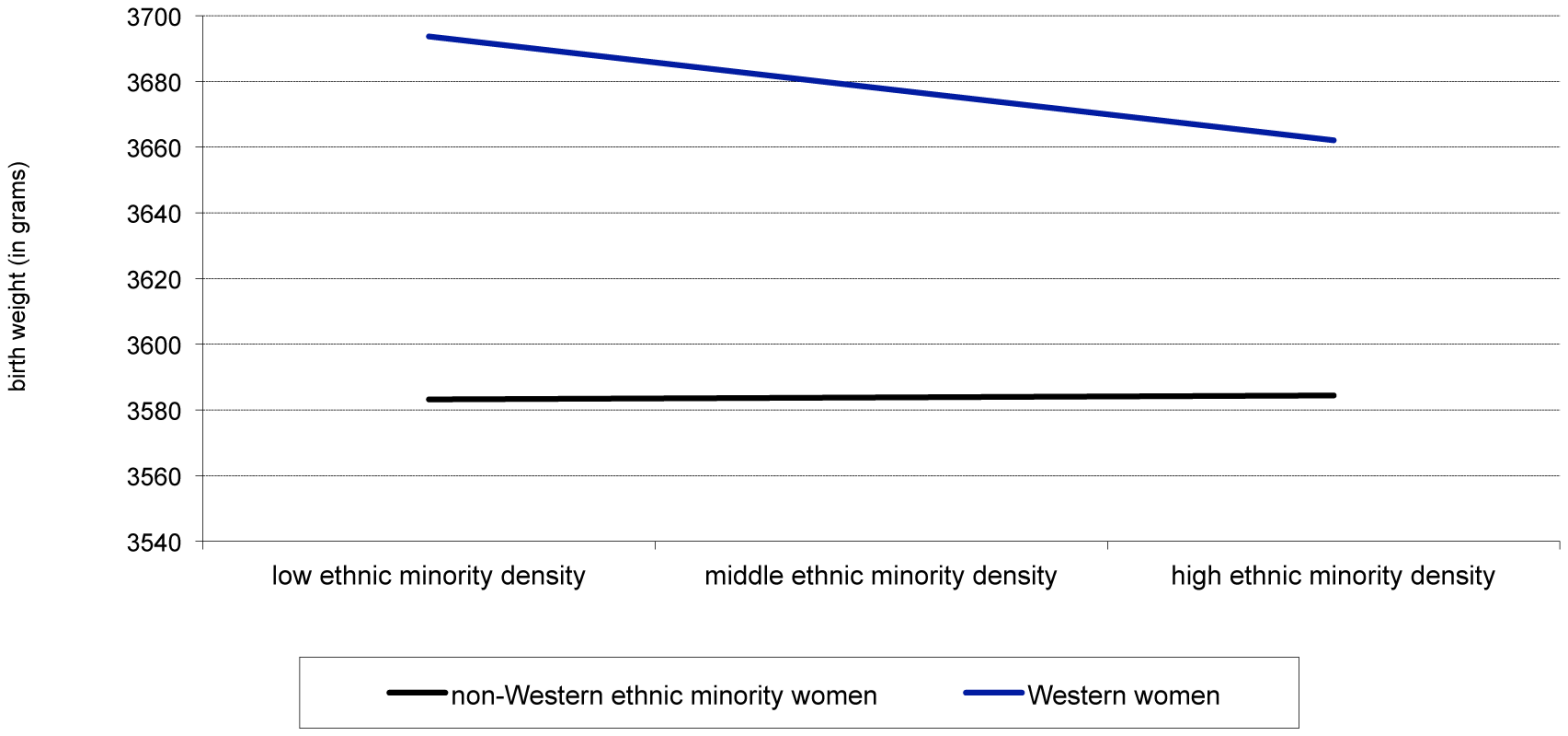
Different association between ethnic minority density and ethnicity. Legend: This figure shows the interaction between increasing levels of ethnic minority density (EMD) at the neighborhood level and the birth weight of infants of Western and non-Western women. The birth weight of infants of Western women decreases with increasing ethnic density; while the birth weight of infants of non-Western women remains stable.

Model 6 shows the results of the interaction term neighborhood ethnic minority density * non-Western ethnicity, which indicates that higher ethnic minority density is associated with higher birth weight for infants of non-Western ethnic minority women as well as reduced risk for prematurity. The full models of both analyses (model 7) show that the effect of ethnic minority density on the outcome variables, as well as the interaction term of ethnic minority density and non-Western ethnicity, remains stable and highly significant. Figure 3 further explores this relationship for birth weight. It is based on values from model 7, where we adjusted for all individual and other neighborhood variables. This figure indicates that the birth weight of infants of Western women decreases as ethnic minority increases, while the birth weight of infants of non-Western women remains stable.

The second models of table 3 and 4 show that neighborhood social capital is associated with increased birth weight, but not with a reduced risk for preterm births. However, the effect of neighborhood social capital becomes attenuated for birth weight after controlling for other neighborhood variables. Additional analyses (results not shown) indicate that ‘feeling of safety’ mediates the relationship between neighborhood social capital and birth weight. By adding the interaction term neighborhood social capital * non-Western ethnicity in model 5, we found that higher neighborhood social capital is associated with higher birth weight of infants among Western women (as compared to non-Western ethnic minority women). We did not find this interaction for preterm births.

### Additional analyses

We ran both analyses mentioned above using the aggregated neighborhood social capital scores instead of the estimate derived from the ecometrics procedure. The beta coefficients and odds ratios of the aggregated social capital scores were slightly higher than those derived from the ecometrics procedure, but the same conclusions can be drawn based on the results. The analysis using ‘small-for-gestational-age’ as the outcome shows that the same conclusions can be drawn from these results as for the birth weight analysis (results for these three additional analyses are not shown).

## Discussion

In line with previous studies, we found a modest but significant clustering of birth weight outcomes and prevalence of prematurity across neighborhoods that is not due to compositional effects. This suggests that the context in which a pregnant woman lives matters for perinatal health in the Netherlands. More specifically, higher ethnic minority density was significantly associated with on average lower birth weight and an increased risk for prematurity, even after controlling for individual and other neighborhood characteristics. Similar to other studies done in English-speaking countries, ethnic minority density had an adverse effect on ethnic majority women, but not on ethnic minority women [16], [23], [24].

The effect size for neighborhood social capital was smaller than that for ethnic density for the birth weight analysis. When controlling for individual and other neighborhood characteristics, neighborhood social capital was significantly associated with higher birth weight for Western women compared to non-Western ethnic minority women (model 5). Buka et al (2003) found that neighborhood social capital was associated with an increase in birth weight of infants of white women, but not of black women in the USA [9]. Interestingly, the latter study did not find an association between ethnic minority density and birth weight. Similar to other studies, we found that ‘feeling of safety’ was associated with increased birth weight in the full model and shown to mediate the association between neighborhood social capital and birth weight. This mediation could explain why the effect of neighborhood social capital was attenuated after the inclusion of feeling of safety in the regression models.

In contrast to the birth weight analysis, neighborhood social capital was not significantly associated with premature birth rates. To our knowledge, no other studies have tested this association. More research is necessary to explore the disparate effect of neighborhood social capital on birth weight and prematurity.

In the fully adjusted models, ‘feeling of safety’ in a neighborhood was associated with increased birth weight and lower risk for small-for-gestational age (OR 0.98, CI 0.97/0.99, results of full analysis not shown) but was *not* associated with risk for prematurity. Two studies in the USA show similar results for the association between a related neighborhood construct, namely ‘crime rate’ and birth weight [10], [16]. Pregnant women living in areas that are perceived to be unsafe tend to show higher levels of stress, which in return has been associated with an increased risk for premature births but possibly less with restricted fetal growth [16], [34]. Therefore, the results of this study are not entirely conclusive. More research needs to be done to investigate how feeling of safety might be associated with birth weight, such as poor dietary patterns or maternal smoking, which are major risk factors for restricted fetal growth.

Studies on neighborhood social capital and ethnic minority density and birth outcomes show comparable effect sizes as our study, with some studies showing slightly larger effect sizes, especially for neighborhood social capital [9], [16], [23]. This might be because some studies involved comparisons between the extreme ranges of exposure, for example comparing very poor neighborhoods to very wealthy neighborhoods. Moreover, it is interesting that this study found an association between neighborhood socioeconomic status and preterm births, whilst the study conducted in Amsterdam, the Netherlands, did not [8].

### Interpretation

#### Ethnicity as a protective factor

The results of this study help explain why two previous studies in the Netherlands [6], [35] found that Western women have higher risks for adverse birth outcomes than non-Western ethnic minority women living in deprived neighborhoods. Our findings show that ethnic minority density is protective for the birth weight of infants and the rate of prematurity of non-Western ethnic minority women. At the same time, neighborhood social capital seems to be slightly protective for the birth weight of infants of Western women. More deprived neighborhoods show higher rates of ethnic minority density and lower levels of neighborhood social capital, hence explaining the relatively disadvantaged position of Western women in these areas.

Most research on the individual determinants of health identifies ‘ethnicity’ (meaning: non-Western ethnic minority status) as only a risk factor for adverse (perinatal) health. This study shows that while ethnic minority status is indeed a risk factor at level 1 (the individual level), it seems to act as a protective factor at level 2 (the neighborhood) in higher ethnic minority density areas. For non-Western ethnic minority women, ethnic minority density seems to mitigate the negative influences of deprived neighborhoods, including lower socioeconomic status, home maintenance, and feeling of safety.

#### Bonding social capital

As stated above, ethnic minority density seems to be protective for non-Western ethnic minority women but not for Western women, and the reverse was partially found for neighborhood social capital. As such, the protective influence of these factors accrue differentially for Western and non-Western ethnic minority women. It is possible that the variable ethnic minority density taps into the bonding social capital of non-Western groups, whilst neighborhood social capital reflects the bonding social capital of (the majority) Western groups. Bonding social capital has been conceptualized as derived from relationships amongst people that share common characteristics such as similar socioeconomic and sociodemographic status [36]. People who are excluded from bonding social capital are typically also excluded from receiving associated benefits.

Indeed, ethnic minority density has often been defined as a proxy for bonding social capital for ethnic minorities [22], [37]. It has been hypothesized that the social capital of a given group increases as it becomes a larger proportion of the total population. At the same time, people who are part of the minority population (and hence a smaller proportion of population) in a neighborhood may face social exclusion and discrimination [22]. This suggests that non-Western ethnic minority women have more access to social capital than Western women in areas of high ethnic minority density in the Netherlands. It should be noted that as we were not able to consider specific non-Western ethnic minority groups in this study, it remains unclear whether the social capital in high ethnic minority density areas are specific to non-Western residents from distinct backgrounds, for example second generation Turkish immigrants or Christian immigrants from Suriname.

The national survey data from which our neighborhood social capital scores were derived may be primarily driven by the perception of majority Western respondents. The survey that provided the neighborhood social capital data is nationally representative, and as such 82.7% of the respondents were Western. We did apply ecometrics when constructing the neighborhood social capital score, which helps to standardize the data, and smooth out variations due to the ethnic (and other) background characteristics of the respondents. However, the resulting score still represents the demographic tendencies of the overall survey sample. If neighborhood social capital measures bonding social capital of Western groups, this could help explain why this index was not associated with better birth outcomes for non-Western women.

### Mechanisms

The mechanisms linking ethnic minority density and neighborhood social capital to birth outcomes remain poorly understood in the literature, and have yet to be investigated in the Netherlands. However, literature on social capital and health provides some suggestions for these mechanisms. Plausibly, these mechanisms also hold true for *bonding* social capital. Social capital has been conceptualized to affect health by: a) promoting the exchange of resources between residents, b) stimulating collective action to improve access to local services and amenities c) enforcing healthy norms of behavior, or conversely exerting informal social control over unhealthy behaviors, and d) facilitating more efficient diffusion of health related information [38], [39].

Applying the above-mentioned mechanisms to the case of premature births, it is possible that bonding social capital improves prevalence of prematurity *directly* by reducing levels of stress [22], [34], for example by reducing exposure to discrimination. Bonding social capital might increase birth weight and reduce prematurity *indirectly* by stimulating healthier pregnancy-related behavior such as reduced maternal smoking or regular visits to prenatal care. More research is necessary to examine if neighborhoods with higher ethnic minority density tend to improve health-related and health care seeking behavior of non-Western ethnic minority women.

### Limitations and strengths

Our study has several limitations. Due to the observational design of our study, we cannot rule out reverse causation, e.g., that poor perinatal health caused lower social capital. Another limitation of the design is that we cannot eliminate bias due to selection into different neighborhoods, meaning that healthy pregnant women move away from low social capital and high ethnic minority density neighborhoods. However, a study showed that selective migration is not a major contributor to health inequalities between neighborhoods in the Netherlands [40]. Another study showed that the vast majority of women who moved during the prenatal phase in the Netherlands remained in neighborhoods of comparable socioeconomic status (and presumably of comparable ethnic minority density and social capital status) [41].

We were not able to control for certain maternal characteristics that have been found to be associated with birth outcomes such as maternal socioeconomic status and smoking during pregnancy [42], [43]. However, several of the individual characteristics we did use (age, parity, ethnicity, and prematurity for the birth weight analysis) are partial proxies for socioeconomic and lifestyle determinants of birth outcomes [5]. We also did not have data on the social capital of individuals from the Perinatal Registration Netherlands data set, which prevented us from testing the cross-level interactions between individual and neighborhood social capital with regards to birth outcomes [44], [45].

Whilst the dichotomous grouping of Western and non-Western women has some major advantages for our analysis, as discussed in the methods section, it is also potentially problematic. This dichotomy lumps together diverse ethnic groups that may differ with respect to patterns of social capital, health behavior, and birth outcomes. Our study is unable to tease out the specific risks of the various ethnic groups. Moreover, the binary construction might also contribute to the perception of all non-Western ethnic groups as being the ‘same’, and reflecting a uniform ethnic minority ‘problem’. This is clearly an oversimplification, and studies on ethnic disparities need to be cognizant of how classifications of ethnicity chosen by researchers might contribute to (mis)conceptions about ethnic groups [46]. We do hope, however, that this study demonstrates that the perinatal health of majority and minority groups should be investigated within specific contexts. In fact, the results of this study indicate the protective effects of ethnicity, in contrast to most studies that underline ethnicity as solely as risk factor [46].

Our assessment of neighborhood social capital did not include questions about perceptions of trust between residents. Some researchers have argued that trust is not an integral part of the construct of social capital, but rather that it arises as a consequence of social interactions between members of a group, i.e. trust is a by-product of social capital, not a constituent part of it [47]. However, as outlined in the introduction, others have put forward that trust is an important psychological resource that lubricates the exchange of favors, acts of voluntarism, and collective action within social networks [48]. In other words, without trust, it would be very difficult to access or mobilize the resources that are embedded within social relations. Hence the fact that our survey did not include an assessment of trust is a limitation. Nonetheless, previous studies have also shown a strong correlation between perceptions of trust and other indicators of social capital, such as informal socializing, reciprocity exchanges, and collective efficacy [49].Thus we believe that the omission of trust in our survey did not introduce a substantial bias in our results.

Despite the above-mentioned limitations, this study also has several strengths. To our knowledge, this is the first study examining neighborhood effects on birth outcomes across an entire country, in this case the Netherlands, as all previous studies are limited to cities or regions. It is also the first study to enquire into the effects of neighborhood social capital and ethnic minority density on birth outcomes in the Netherlands. Moreover, it is one of the few studies to examine the association of a range of both physical (home maintenance, urbanity) and social (ethnic density, neighborhood social capital, feeling of safety, socioeconomic status) neighborhood characteristics on birth outcomes. Another strength of this study is that it is one of the few to use a neighborhood social capital score derived via an ecometrics procedure, which improves reliability.

### Public health implications

We found modest but significant effects of neighborhood level characteristics on average birth weight and risk for premature births. As such, policies targeting change at the neighborhood level have the potential to affect birth outcomes across entire neighborhoods. We recommend future research into the cost-effectiveness of interventions targeting change at the neighborhood versus the individual level (or both). Moreover, we suggest future studies that are able to incorporate more specific ethnic categories, for instance by using country of birth of pregnant women, and that of her father/mother, or by using self-ascribed ethnicity.

## Supporting Information

Table S1 **Descriptive statistics of sources of data.**
(DOCX)

## References

[1] EURO-PERISTAT project with SCPE E (2013) European perinatal health report. The health and care of pregnant women and babies in Europe in 2010.

[2] de Graaf JP, Steegers EA, Bonsel GJ (2013) Inequalities in perinatal and maternal health. Current Opinion in Obstetrics and Gynecology 25: 98–108. 23425665 10.1097/GCO.0b013e32835ec9b0

[3] Poeran J, Denktas S, Birnie E, Bonsel GJ, Steegers EAP (2011) Urban perinatal health inequalities. Journal of Maternal-Fetal and Neonatal Medicine 24: 643–646. 20836740 10.3109/14767058.2010.511341

[4] Goedhart G, Van Eijsden M, Van Der Wal MF, Bonsel GJ (2008) Ethnic differences in preterm birth and its subtypes: the effect of a cumulative risk profile. BJOG: An International Journal of Obstetrics & Gynaecology 115: 710–719. 18410654 10.1111/j.1471-0528.2008.01682.x

[5] de Graaf JP, Ravelli ACJ, de Haan MAM, Steegers EAP, Bonsel GJ (2012) Living in deprived urban districts increases perinatal health inequalities. Journal of Maternal-Fetal and Neonatal Medicine: 1–29. 10.3109/14767058.2012.735722 23039164

[6] Poeran J, Maas AF, Birnie E, Denktas S, Steegers EA, et al.. (2013) Social deprivation and adverse perinatal outcomes among Western and non-Western pregnant women in a Dutch urban population. Social Science & Medicine. 10.1016/j.socscimed.2013.02.008 23465203

[7] Mook-Kanamori DO, Steegers EA, Eilers PH, Raat H, Hofman A, et al (2010) Risk factors and outcomes associated with first-trimester fetal growth restriction. JAMA: The Journal of the American Medical Association 303: 527–534. 20145229 10.1001/jama.2010.78

[8] Agyemang C, Vrijkotte T, Droomers M, Van der Wal M, Bonsel G, et al (2009) The effect of neighbourhood income and deprivation on pregnancy outcomes in Amsterdam, The Netherlands. Journal of epidemiology and community health 63: 755–760. 19679715 10.1136/jech.2008.080408

[9] Buka SL, Brennan RT, Rich-Edwards JW, Raudenbush SW, Earls F (2003) Neighborhood support and the birth weight of urban infants. American journal of epidemiology 157: 1–8. 12505884 10.1093/aje/kwf170

[10] Morenoff JD (2003) Neighborhood mechanisms and the spatial dynamics of birth weight1. American Journal of Sociology 108: 976–1017. 10.1086/374405 14560732

[11] Pearl M, Braveman P, Abrams B (2001) The relationship of neighborhood socioeconomic characteristics to birthweight among 5 ethnic groups in California. Journal Information 91.. 10.2105/ajph.91.11.1808 PMC1446884 11684609

[12] Messer LC, Vinikoor LC, Laraia BA, Kaufman JS, Eyster J, et al (2008) Socioeconomic domains and associations with preterm birth. Social Science & Medicine 67: 1247–1257. 18640759 10.1016/j.socscimed.2008.06.009

[13] O'Campo P, Burke JG, Culhane J, Elo IT, Eyster J, et al (2008) Neighborhood deprivation and preterm birth among non-Hispanic Black and White women in eight geographic areas in the United States. American journal of epidemiology 167: 155–163. 17989062 10.1093/aje/kwm277

[14] Pickett KE, Ahern JE, Selvin S, Abrams B (2002) Neighborhood socioeconomic status, maternal race and preterm delivery: a case-control study. Annals of epidemiology 12: 410–418. 12160600 10.1016/s1047-2797(01)00249-6

[15] Collins J Jr, David RJ (1997) Urban violence and African-American pregnancy outcome: an ecologic study. Ethnicity & disease 7: 184. 9467700

[16] Masi CM, Hawkley LC, Harry Piotrowski Z, Pickett KE (2007) Neighborhood economic disadvantage, violent crime, group density, and pregnancy outcomes in a diverse, urban population. Social Science & Medicine 65: 2440–2457. 17765371 10.1016/j.socscimed.2007.07.014

[17] Coleman JS, Coleman JS (1994) Fondations of Social Theory: Harvard University Press.

[18] Putnam RD (1993) The prosperous community. The american prospect 4: 35–42.

[19] Lochner KA, Kawachi I, Brennan RT, Buka SL (2003) Social capital and neighborhood mortality rates in Chicago. Social Science and Medicine 56: 1797–1806. 12639596 10.1016/s0277-9536(02)00177-6

[20] Mohnen SM, Groenewegen PP, Völker B, Flap H (2011) Neighborhood social capital and individual health. Social Science & Medicine 72: 660–667. 21251743 10.1016/j.socscimed.2010.12.004

[21] van Hooijdonk C, Droomers M, Deerenberg IM, Mackenbach JP, Kunst AE (2008) The diversity in associations between community social capital and health per health outcome, population group and location studied. International Journal of Epidemiology 37: 1384–1392. 18782895 10.1093/ije/dyn181

[22] Pickett KE, Wilkinson RG (2008) People like us: ethnic group density effects on health. Ethnicity & health 13: 321–334. 18701992 10.1080/13557850701882928

[23] Roberts EM (1997) Neighborhood social environments and the distribution of low birthweight in Chicago. American journal of public health 87: 597–603. 9146438 10.2105/ajph.87.4.597 PMC1380839

[24] Auger N, Daniel M, Platt RW, Wu Y, Luo ZC, et al (2008) Association between perceived security of the neighbourhood and small-for-gestational-age birth. Paediatric and perinatal epidemiology 22: 467–477. 18782253 10.1111/j.1365-3016.2008.00959.x

[25] Grady SC (2006) Racial disparities in low birthweight and the contribution of residential segregation: a multilevel analysis. Social science & medicine (1982) 63: 3013. 16996670 10.1016/j.socscimed.2006.08.017

[26] Van Huijsduijnen JH, Van Til RJ, Verhoog E, Gopal K, Ferment B, et al.. (2007) WoON 2006 Onderzoeksdocumentatie e Module Woningmarkt. [WoOn 2006 documentation of the housing demand survey e Module housing market]. Delft: The Netherlands Ministry of Housing, Spatial Planning and Environment [Ministerie van VROM].

[27] Barker DJP (2006) Adult consequences of fetal growth restriction. Clinical obstetrics and gynecology 49: 270–283. 16721106 10.1097/00003081-200606000-00009

[28] Moster D, Lie RT, Markestad T (2008) Long-term medical and social consequences of preterm birth. New England Journal of Medicine 359: 262–273. 18635431 10.1056/NEJMoa0706475

[29] Marlow N, Wolke D, Bracewell MA, Samara M (2005) Neurologic and developmental disability at six years of age after extremely preterm birth. New England Journal of Medicine 352: 9–19. 15635108 10.1056/NEJMoa041367

[30] Raudenbush SW, Sampson RJ (1999) Ecometrics: toward a science of assessing ecological settings, with application to the systematic social observation of neighborhoods. Sociological methodology 29: 1–41.

[31] Mujahid MS, Roux AVD, Morenoff JD, Raghunathan T (2007) Assessing the measurement properties of neighborhood scales: from psychometrics to ecometrics. American journal of epidemiology 165: 858–867. 17329713 10.1093/aje/kwm040

[32] Hox J (2002) Multilevel analysis: Techniques and applications: Mahwah, NJ: Lawrence Erlbaum Associates.

[33] Snijders T, Bosker R (1999) Multilevel modeling: An introduction to basic and advanced multilevel modeling.

[34] Dole N, Savitz DA, Hertz-Picciotto I, Siega-Riz AM, McMahon MJ, et al (2003) Maternal stress and preterm birth. American journal of epidemiology 157: 14–24. 12505886 10.1093/aje/kwf176

[35] de Graaf JP, Ravelli AC, Visser GH, Hukkelhoven C, Tong WH, et al (2010) Increased adverse perinatal outcome of hospital delivery at night. BJOG 117: 1098–1107. 20497413 10.1111/j.1471-0528.2010.02611.x

[36] Szreter S, Woolcock M (2004) Health by association? Social capital, social theory, and the political economy of public health. International Journal of Epidemiology 33: 650–667. 15282219 10.1093/ije/dyh013

[37] McKenzie K, Whitley R, Weich S (2002) Social capital and mental health. The British Journal of Psychiatry 181: 280–283. 12356653 10.1192/bjp.181.4.280

[38] Berkman LF, Kawachi I (2000) Social cohesion, social capital, and health. In: Social epidemiology Berkman LF, Kawachi I, eds. Social epidemiology. New York: Oxford University Press 2000: 174–90.

[39] Lin N (2002) Social capital: A theory of social structure and action: Cambridge University Press.

[40] Van Lenthe F, Mackenbach J (2002) Neighbourhood deprivation and overweight: the GLOBE study. International journal of obesity and related metabolic disorders: journal of the International Association for the Study of Obesity 26: 234. 10.1038/sj.ijo.0801841 11850756

[41] Reitsma J, Kardaun J, Gevers E, De Bruin A, Van der Wal J, et al (2003) [Possibilities for anonymous follow-up studies of patients in Dutch national medical registrations using the Municipal Population Register: a pilot study]. Nederlands tijdschrift voor geneeskunde 147: 2286. 14655296

[42] Jaddoe VW, Troe EJW, Hofman A, Mackenbach JP, Moll HA, et al (2008) Active and passive maternal smoking during pregnancy and the risks of low birthweight and preterm birth: the Generation R Study. Paediatric and perinatal epidemiology 22: 162–171. 18298691 10.1111/j.1365-3016.2007.00916.x

[43] Kramer MS, Séguin L, Lydon J, Goulet L (2000) Socio-economic disparities in pregnancy outcome: why do the poor fare so poorly? Paediatric and perinatal epidemiology 14: 194–210. 10949211 10.1046/j.1365-3016.2000.00266.x

[44] Moore S, Haines V, Hawe P, Shiell A (2006) Lost in translation: a genealogy of the “social capital” concept in public health. Journal of epidemiology and community health 60: 729–734. 16840764 10.1136/jech.2005.041848 PMC2588078

[45] Poortinga W (2006) Social relations or social capital? Individual and community health effects of bonding social capital. Social science & medicine (1982) 63: 255. 16427171 10.1016/j.socscimed.2005.11.039

[46] Proctor A, Krumeich A, Meershoek A (2011) Making a difference: The construction of ethnicity in HIV and STI epidemiological research by the Dutch National Institute for Public Health and the Environment (RIVM). Social Science & Medicine 72: 1838–1845. 21601970 10.1016/j.socscimed.2011.03.043

[47] Harpham T, Grant E, Thomas E (2002) Measuring social capital within health surveys: key issues. Health policy and planning 17: 106–111. 11861592 10.1093/heapol/17.1.106

[48] Kawachi I (2010) Social Capital and Health. In: Handbook of medical sociology Bird CE, Fremont AM, Zimmermans S, eds. Handbook of medical sociology. Nashville, TN: Vanderbilt University Press 2010: 18–32.

[49] Sampson RJ, Raudenbush SW, Earls F (1997) Neighborhoods and violent crime: A multilevel study of collective efficacy. Science 277: 918–924. 9252316 10.1126/science.277.5328.918

