# Supplementary material for: The Association of Neighborhood Social Capital and Ethnic (Minority) Density with Pregnancy Outcomes in the Netherlands
Source: PLoS One. 2014 May 7;9(5):e95873. doi: 10.1371/journal.pone.0095873 (PMC4012999; doi:10.1371/journal.pone.0095873)
Supplement: Table S1 — Descriptive statistics of sources of data. (DOCX) [file pone.0095873.s001.docx]

**Appendix - supplementary information**

**Table S1. Descriptive statistics of sources of data**

|  | **Perinatal Registration Netherlands (PRN)** | **Statistics Netherlands (CBS)** | **Housing and Living Survey (WoON)** | **The Netherlands Institute for Social Research (SCP)** |
| --- | --- | --- | --- | --- |
| **Individuals in sample** | 1,620,315 | 16,328,160 | 64,005 | 100,000 |
| **Number of neighborhoods (4-digit zipcode area)** | 5454 | 3981 | 3495 | 3469 |
| **Average number of individuals per neighborhood** | 295.8 | 4080 | 18.3 | 100 |
| **Year of data collection** | 2000-2008 | 2006 | 2005-2006 | 2002-2006 |
| **Sampling procedure** | Clinical registration | Civic registration | Random sample | Random sample |
| **Response rate** | 97% | N/A | 56% | 25% |
